# Early and late initiation of the Ponseti method yield comparable outcomes in congenital idiopathic clubfoot: a systematic review and meta-analysis

**DOI:** 10.1051/sicotj/2025071

**Published:** 2026-02-26

**Authors:** Abdullah Addar, Abdullah I. Alturki, Yazeed Alsanad, Turki Alotaibi, Fahad Alshayhan

**Affiliations:** 1 Department of Orthopedic Surgery, College of Medicine, King Saud University P.O. Box 2925 Riyadh 11461 Saudi Arabia; 2 College of Medicine, King Saud University P.O. Box 2925 Riyadh 11461 Saudi Arabia

**Keywords:** Age of initiation, Ponseti method, Number of casts, Relapse rate, Idiopathic clubfoot, Systematic review, Meta analysis

## Abstract

*Introduction*: The optimal timing to initiate the Ponseti method for congenital idiopathic clubfoot remains uncertain. This systematic review and meta-analysis aimed to evaluate whether starting treatment within the first four weeks of life improves outcomes compared to later initiation. *Methods*: Following PRISMA guidelines (PROSPERO ID: CRD42025650117), MEDLINE, Embase, Cochrane Library, and Google Scholar were searched for studies comparing early (≤4 weeks) versus late (>4 weeks) initiation of the Ponseti method. Outcomes included the number of casts, the relapse rate, and the need for tenotomy. Data were pooled using a random-effects model, and study quality was assessed using the MINORS tool. *Results*: Six studies involving 467 patients (689 feet) met the inclusion criteria. Early initiation was associated with a slightly higher mean number of casts (MD = 0.72, 95% CI [0.33–1.10], *p* = 0.0002), but this difference was not significant in the overall pooled analysis (MD = 0.06, 95% CI [−1.08–1.21], *p* = 0.91). Relapse (OR = 0.70, *p* = 0.68) and tenotomy rates (OR = 0.68, *p* = 0.41) were comparable between groups. *Discussion*: Although earlier treatment may require more casts, it does not reduce relapse or tenotomy rates. These findings suggest that initiating treatment after four weeks yields comparable outcomes, offering flexibility in clinical practice without compromising results. Variability across studies highlights the need for standardized treatment protocols and well-designed randomized controlled trials to confirm the optimal initiation age.

## Introduction

Clubfoot, or congenital talipes equinovarus (CTEV), is a common congenital orthopedic deformity characterized by forefoot adduction, midfoot cavus, hindfoot equinus, and hindfoot varus [1]. It affects approximately one to two per 1,000 live births, with a male predominance of nearly 3:1, and about half of the cases present bilaterally [2]. The Ponseti method remains the gold standard for managing idiopathic clubfoot and consists of serial manipulations and casting, followed by percutaneous Achilles tenotomy and subsequent bracing to maintain correction [3]. Long-term analyses have shown that Ponseti-treated feet achieve near-normal size and function, confirming the method’s durable anatomical and cosmetic success [4]. The timing of Ponseti treatment initiation has been proposed as a key determinant of treatment success. Early initiation has traditionally been encouraged under the assumption that neonatal tissues are more pliable and easier to manipulate [3, 5]. Recent literature has highlighted the potential benefits of starting treatment before four weeks of life, suggesting that earlier intervention may yield improved outcomes in terms of treatment complexity and relapse rates [6]. However, other reports suggest that initiating treatment after four weeks of age may be associated with comparable or even improved outcomes, possibly due to greater tissue stiffness reducing over-correction and better caregiver compliance [7]. Current guidelines do not provide a definitive recommendation on the ideal age for treatment initiation, leading to variability in clinical practice. In response to these uncertainties, this systematic review and meta-analysis aims to clarify the relationship between the age of initiation of the Ponseti method and treatment outcomes. We seek to establish whether starting treatment before or after four weeks significantly affects the number of casts, the overall relapse rate, and the need for Achilles tenotomy.

## Materials and methods

This systematic review and meta-analysis were conducted in accordance with PRISMA guidelines and registered on PROSPERO (ID: CRD42025650117) [8]. A comprehensive literature search was performed in December 2024 across MEDLINE, Embase, Cochrane Library, and Google Scholar. The search combined keywords for clubfoot (e.g., congenital talipes equinovarus, idiopathic clubfoot), Ponseti method (including Ponseti technique or serial casting), and treatment initiation age (e.g., age of treatment, when to treat). No date or publication status restrictions were applied. Reference lists of relevant articles were also screened to ensure comprehensive coverage.

Studies were eligible if they (a) involved infants under 1 year of age diagnosed with idiopathic (isolated) clubfoot (i.e., no syndromic or neuromuscular etiology). (b) Utilized the Ponseti treatment protocol for clubfoot correction. The Ponseti method involves weekly gentle manipulations and serial casts, typically followed by a percutaneous Achilles tenotomy and bracing to maintain correction. (c) Included a direct comparison between early (treatment initiated at age ≤ 4 weeks) versus late (initiated > 4 weeks of age) Ponseti treatment. (d) A four-week age cutoff was chosen as it is the most common threshold in the literature. (e) Reported at least one of the key outcomes of interest, primarily the number of casts required, relapse rate, and need for tenotomy. (f) Randomized controlled trials, cohort studies, case-control studies, cross-sectional studies, or large case series suitable for comparison.

Studies were excluded if they: (a) included patients with syndromic or non-idiopathic clubfoot (e.g. arthrogryposis or spina bifida), (b) were not published in English, (c) had significant methodological flaws or did not report relevant outcomes, (d) were case reports, conference abstracts, or animal studies, or (e) involved infants who had received prior treatment for clubfoot before Ponseti casting. Studies meeting the inclusion criteria on population and intervention but without separable data for early vs. late initiation were also excluded from the meta-analysis.

**Figure 1:**
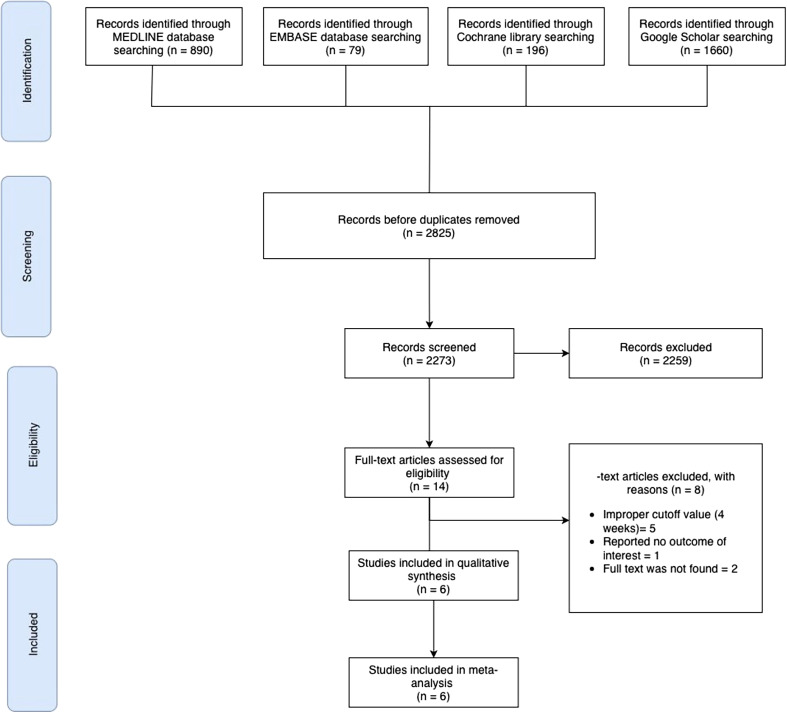
PRISMA flow chart of the selection process.

All titles and abstracts retrieved were independently screened by two reviewers using the Rayyan web application for systematic reviews [9]. Duplicates and clearly irrelevant articles were removed. The full texts of potentially eligible studies were then assessed in detail by the reviewers, with any disagreements resolved through discussion by all co-authors. The study selection process is summarized in a PRISMA flow diagram (Figure 1). The initial search yielded 2,825 records; after removing duplicates, 2,273 unique records were screened, and 2,259 were excluded based on title/abstract. Fourteen articles underwent full-text evaluation, of which six studies met all eligibility criteria and were included in the review [6, 7, 10–13].

The six included studies comprised a total of 467 patients (689 affected feet). Of these, 137 patients began Ponseti treatment at ≤4 weeks of age, and 280 began after 4 weeks. Across studies, the age at treatment initiation ranged from as early as 2 days old up to 23 months. All cases were idiopathic clubfoot, and all studies employed the standard Ponseti method as described above. Four of the studies documented follow-up periods, which ranged from 12 months to 48 months post-treatment, allowing assessment of mid-term outcomes [7, 10, 12, 13]. Five studies reported the sex of the patients; 308 were males, and 109 were females [7, 10–13]. Peshang et al. [12] study was included in the systematic review, not a meta-analysis, due to insufficient data for the analysis. Full details about the characteristics of the included studies are available in Table 1.

**Table 1:** Characteristics of the included studies.

Study ID	Study design	Year of publication	Country of origin	Total number of patients included	Number of patients LESS than 4 weeks	Number of patients MORE than 4 weeks	Age range	Mean Age in weeks (days), SD	Sex, Male (N)	Sex, Female (N)	Left foot (N)	Right foot (N)	Bilateral foot (N)	Total Number of feet (N)	Follow-up duration in months
Lee et al., 2020 [10]	Retrospective study	2020	Malaysia	54	21	33	1–23 months	Group 1: 1.8 weeks, Group 2: 26.2 weeks	35	19	N/A	N/A	23	77	22.39
Vaishy et al., 2020 [11]	Retrospective study	2020	India	200	38	162	N/A	N/A	149	51	49	54	97	297	N/A
Khurana et al., 2024 [6]	Prospective Cohort Study	2024	India	50	N/A	N/A	N/A	2.66 ± 2.79 (months)	46 (feet)	30 (feet)	N/A	N/A	N/A	76	N/A
Liu et al., 2018 [7]	Retrospective study	2018	China	90	30	60	2 days to 6 months	48 days	72	18	N/A	N/A	41	131	48
Peshang et al., 2024 [12]	Prospective cohort study	2024	Kurdistan	44	29	15	3 days to 5 months	N/A	30	14	31	38	24	68	12
Iltar et al., 2010 [13]	Prospective cohort study	2010	Turkey	29	19	10	2 days to 11 months	59 days	22	7	N/A	N/A	11	40	34.1

Data from each included study were extracted independently by two reviewers using a standardized form. Extracted variables included publication details (year, journal, country), study design, sample size, patient demographics (age at treatment initiation, sex, laterality of clubfoot), and clinical details such as follow-up duration and specifics of the Ponseti treatment protocol. Key outcome measures were collected for both the early-initiation and late-initiation groups: the mean number of casts required to achieve correction, relapse rate (as defined by the need for further intervention), and the proportion of cases undergoing Achilles tenotomy. All data entries were cross-checked by a second reviewer, and disagreements were resolved by discussion.

The risk of bias and methodological quality of the included non-randomized studies were assessed using the Methodological Index for Non-Randomized Studies (MINORS) checklist [14]. This tool evaluates 12 methodological domains, each scored 0 (not reported), 1 (reported but inadequate), or 2 (reported and adequate), with a maximum of 24 points for comparative studies, and 16 for non-comparative studies. Two reviewers independently appraised each study using the MINORS criteria; any discrepancies in scoring were discussed and resolved by involving a third reviewer if necessary. The overall quality of studies was taken into account when interpreting the results. All included studies were found to be of moderate-to-high quality based on MINORS, with no critical risk of bias identified (see Table 2 for detailed scores).

**Table 2:** Shows the Bias Assessment MINORS assessment tool.

Study ID	Study Design	1) A clearly stated aim	2) Inclusion of consecutive patients	3) Prospective collection of data	4) Endpoints appropriate to the aim of the study	5) Unbiased assessment of the study endpoint	6) Follow-up period appropriate to the aim of the study	7) Loss to follow-up less than 5%	8) Prospective calculation of the study size	9) An adequate control group (Comparative studies only)	10) Contemporary groups (Comparative studies only)	11) Baseline equivalence of groups (Comparative studies only)	12) Adequate statistical analyses (Comparative studies only)	Total Score
Lee et al., 2020 [10]	Retrospective study	2	2	2	2	2	1	1	1	N/A	N/A	N/A	N/A	13/16
Vaishy et al., 2020 [11]	Retrospective study	2	2	2	2	1	1	1	0	N/A	N/A	N/A	N/A	11/16
Khurana et al., 2024 [6]	Prospective Cohort Study	2	2	2	2	2	1	1	1	N/A	N/A	N/A	N/A	12/16
Liu et al., 2018 [7]	Retrospective study	2	2	2	2	2	2	2	0	N/A	N/A	N/A	N/A	14/16
Peshang et al., 2024 [12]	Prospective cohort study	2	2	2	2	2	1	1	2	N/A	N/A	N/A	N/A	14/16
Iltar et al., 2010 [13]	Prospective cohort study	2	2	2	2	1	1	1	1	N/A	N/A	N/A	N/A	12/16

Pooled analyses were performed using Review Manager (RevMan, Version 5.4.1) [15]. For continuous outcomes (such as the number of casts), mean differences (MD) with corresponding 95% confidence intervals (CI) were calculated under a random-effects model to account for expected inter-study heterogeneity. For dichotomous outcomes (such as relapse and tenotomy rates), pooled odds ratios (OR) with 95% CI were computed. All meta-analyses employed the inverse-variance weighting approach. Statistical significance was defined at the *p* < 0.05 level. Heterogeneity among studies was quantified using the *I*^2^ statistic. In cases of substantial heterogeneity (*I*^2^ > 50%), potential sources were explored by performing a leave-one-out sensitivity analysis (recalculating the meta-analysis, omitting one study at a time). This approach assessed the stability of the results and the influence of any single study on the pooled estimates. Results of the meta-analysis are presented as forest plots, and publication bias was assessed via funnel plot inspection when ≥10 studies were available (not applicable in this review due to the small number of studies).

## Results

Early and late initiation of the Ponseti method required a similar number of casts on average. A meta-analysis of four studies (Table 3) found no significant difference in cast count between infants who began treatment at ≤ 4 weeks old and those who began after 4 weeks (pooled mean difference [MD] = 0.06, 95% confidence interval [CI] −1.08 to 1.21; *p* = 0.91). Between-study heterogeneity was substantial (*I*^2^ = 91%). However, a leave-one-out sensitivity analysis indicated that the result was influenced by a single study; excluding the study by Khurana et al. [6] reduced heterogeneity to 0% and revealed that early initiation might require slightly more casts (MD = 0.72, 95% CI 0.33–1.10; *p* = 0.0002) (Table 4).

**Table 3:** Meta-analysis of the mean number of casts. 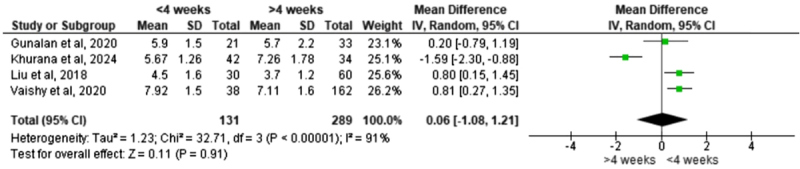

**Table 4:** Leave-one-out meta-analysis of the mean number of casts. 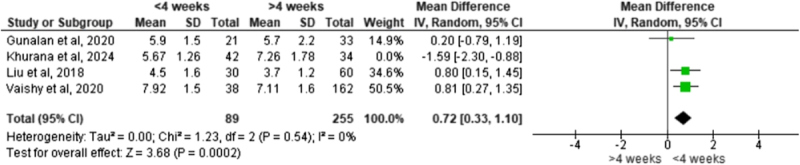

Relapse rates did not differ significantly between early and late initiation groups. In a meta-analysis of three studies (Table 5), early treatment showed no significant reduction in relapse risk compared to later treatment (pooled odds ratio [OR] = 0.70, 95% CI 0.13–3.88; *p* = 0.68). Heterogeneity was moderate-to-high (*I*^2^ = 51%). A leave-one-out analysis removing the study by Liu et al. [7] eliminated the heterogeneity (*I*^2^ = 0%), without materially changing the pooled effect, confirming that early initiation offers no clear advantage in terms of relapse rate.

**Table 5:** Meta-analysis of the relapse rate. 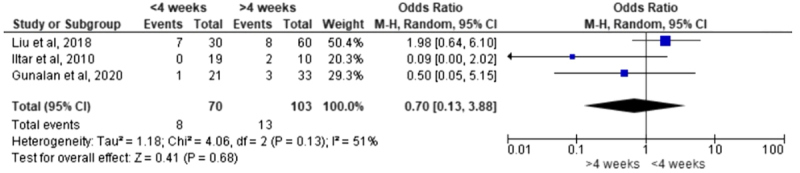

The need for Achilles tenotomy was also comparable between early and late initiation. A two-study meta-analysis (Table 6) indicated no significant difference in tenotomy rates between the groups (pooled OR = 0.68, 95% CI 0.27–1.71; *p* = 0.41). Heterogeneity was moderate (*I*^2^ = 43%), and no further sensitivity analysis was feasible given the limited number of studies for this outcome.

**Table 6:** Meta-analysis of tenotomies. 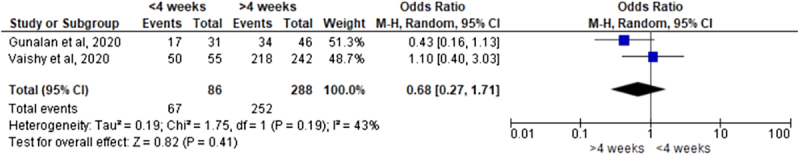

Overall, six studies (*n* = 467 patients, affecting 689 clubfeet) met the inclusion criteria for this review (Figure 1; Table 1). Among the five studies that reported age-stratified data, 137 infants began Ponseti casting at ≤ 4 weeks of age, and 280 infants began after 4 weeks. All included studies were judged to be of high quality, with a low risk of bias according to the MINORS assessment (Table 2).

## Discussion

Idiopathic congenital clubfoot is a common pediatric deformity typically managed with the Ponseti method [1, 3], and the optimal timing for initiating treatment remains debated [5, 7, 13]. This systematic review and meta-analysis compared early (≤4 weeks) versus late (>4 weeks) initiation of Ponseti casting and found no significant differences in the primary clinical outcomes. Across six studies including 467 infants, the number of casts required, the rate of relapse, and the need for Achilles tenotomy were comparable between early and late treatment groups [6, 7, 10–13], indicating similar overall correction success in both cohorts.

In this study, several limitations must be acknowledged. The evidence is based on six observational cohorts rather than randomized trials, introducing potential bias and confounding despite moderate overall study quality. Considerable heterogeneity existed, particularly in the number of casts (*I*^2^ > 90%), likely due to differences in patient characteristics and treatment protocols. Important variables such as initial deformity severity (e.g., Pirani or Dimeglio scores) and bracing compliance were inconsistently reported, limiting our ability to assess their influence on outcomes [16, 17]. Follow-up durations were relatively short (1–4 years), which may underestimate late relapses, and the findings may not generalize to atypical or syndromic clubfoot. Restricting the search to English-language studies also raises the possibility of publication bias. These limitations warrant cautious interpretation, and future prospective or randomized studies with standardized severity scoring and compliance monitoring are needed to better define the optimal timing of Ponseti treatment.

Across the included studies, beginning Ponseti casting at ≤4 weeks did not significantly reduce the number of casts required compared with initiation after 4 weeks. Infants treated in an early period required a similar number of casts to those who started later, with pooled analyses showing no meaningful difference between groups [6, 7, 10, 11]. Some studies even reported a slight trend toward more casts in the early-start group, although this was inconsistent and not statistically significant. These findings align with Alves et al., who also found no significant difference in casting requirements based on treatment timing [18], supporting the conclusion that the initial correction phase is not strongly influenced by the infant’s age at treatment onset.

Relapse rates were similar between early and late treatment groups, with no statistically significant differences identified across the included relevant studies [7, 10, 13]. This indicates that relapse is more closely related to factors such as initial deformity severity and long-term brace adherence rather than the timing of initial casting. Dobbs et al. identified brace compliance (not treatment age) as the major predictor of relapse [19], while Morcuende et al. demonstrated that strict adherence to the Ponseti protocol markedly reduces the need for later corrective procedures [20]. Khurana et al. further reported that infants treated between 1 and 3 months of age had some of the lowest relapse rates [6], suggesting that immediate neonatal initiation offers no proven long-term advantage.

The proportion of infants requiring a percutaneous Achilles tenotomy did not differ significantly between early and late initiation groups. Similar tenotomy rates were reported across included relevant studies [10, 11], consistent with the expectation that most idiopathic clubfeet require tenotomy to correct residual equinus. This finding aligns with foundational Ponseti principles, which emphasize that achieving adequate dorsiflexion often necessitates tenotomy regardless of the age at which casting begins [3, 21]. The current evidence, therefore, suggests that treatment timing does not influence the inherent likelihood of requiring this standard component of the Ponseti protocol.

Although the Ponseti method has become the gold standard for management due to its high success rates and minimally invasive approach [4, 22], several challenges continue to impact treatment outcomes. These include variability in initial deformity severity, differences in clinical expertise, the technical difficulty of casting very young infants, and, most importantly, long-term brace adherence, which remains the strongest predictor of relapse [19]. In addition, late presentation, limited parental understanding of bracing protocols, and inconsistent severity scoring across studies and clinical settings contribute to heterogeneity in reported outcomes [16, 17, 23].

The appropriate timing for initiating treatment has long been debated. Historically, very early neonatal casting was favored because the newborn foot is more flexible and theoretically easier to mold [3, 5]. However, accumulating evidence suggests that the Ponseti method is highly effective throughout early infancy and that immediate treatment does not necessarily yield superior long-term outcomes [6, 7, 10, 11, 13]. Some studies report improved correction and lower relapse rates when treatment begins between 4 and 12 weeks of age, likely due to larger foot size, reduced overcorrection risk, and improved caregiver readiness for ongoing brace maintenance [6, 7, 13]. These findings support a more flexible, individualized approach that considers infant size, parental capacity, and clinic accessibility when determining the start of treatment.

There are advantages and disadvantages to both early and later initiation of Ponseti casting. Early neonatal treatment offers the benefit of high tissue plasticity and rapid molding potential [3, 5], but is technically challenging due to small foot size and may increase the risk of overcorrection when tissues are exceptionally pliable [13]. Parental fatigue or logistical difficulties during the immediate postpartum period can also negatively influence bracing compliance. Conversely, initiating treatment slightly later (after several weeks) may facilitate more precise casting, allow caregivers to better adapt to follow-up demands, and reduce the likelihood of overcorrection due to mild age-related stiffness [6, 7, 10]. Importantly, excessively delaying treatment beyond early infancy remains inadvisable, as the deformity becomes progressively rigid with age [22].

Overall, the intrinsic characteristics of the clubfoot deformity, combined with practical challenges in severity assessment and brace adherence, suggest that the timing of Ponseti initiation should be individualized within the early infancy window. The available evidence supports that treatment can begin anytime within the first few months of life without compromising correction, provided that casting is performed correctly and bracing is rigorously maintained.

In conclusion, our systematic review and meta-analysis indicate that initiating the Ponseti method at or beyond four weeks of age yields clinical outcomes comparable to initiation in the neonatal period, with no clear benefit to very early casting in terms of cast number, relapse risk, or tenotomy need. These findings support a more flexible approach to timing, allowing clinicians to commence treatment within the first few months of life based on practical considerations without compromising effectiveness, while underscoring the continued importance of adherence to the Ponseti protocol to ensure successful long-term correction.

## Data Availability

Data associated with this article are available in an Excel sheet and ready to share when necessary.
